# A new survey to evaluate conflict of interest policies at academic medical centers

**DOI:** 10.1371/journal.pone.0172472

**Published:** 2017-03-15

**Authors:** Marcia Hams, Wells G. Wilkinson, Lynn Zentner, Cory Schmidt, Raed A. Dweik, Matthew Karafa, Susannah L. Rose

**Affiliations:** 1 Community Catalyst, Boston, MA, United States of America; 2 Office of Institutional Compliance at the University of Minnesota, Minneapolis, MN, United States of America; 3 Innovation Management and Conflict of Interest (IM&COI) Program at Cleveland Clinic, Cleveland, OH, United States of America; 4 Cleveland Clinic Lerner College of Medicine of Case Western Reserve University, Cleveland, OH, United States of America; 5 Department of Quantitative Health Sciences, Cleveland Clinic, Cleveland, OH, United States of America; 6 Office of Patient Experience, Department of Bioethics, at Cleveland Clinic, Cleveland, OH, United States of America; York University, CANADA

## Abstract

**Background:**

A majority of academic medical centers (AMCs) have now adopted conflict of interest policies (COI) to address relationships with pharmaceutical and device industries that can increase the risk of bias in patient care, education and research. However, AMCs may have little information on the impact of their policies. This paper provides a new method, which is a free, publicly-available survey, to fill this information gap and improve COI programs at AMCs.

**Methods & findings:**

The survey, piloted in three AMCs and designed in collaboration with national conflicts of interest policy experts, covers a range of universal compliance-related concerns, which allows institutions to tailor questions to align with their own policies and culture. The survey was low-burden, and provided important data for these AMCs to evaluate their policies. A descriptive analysis of the pooled pilot site data (n = 1578) was performed, which found that a majority of respondents did not have financial ties with industry and a majority was satisfied with specific COI policies at their institutions. The analysis also showed that the survey is sensitive to differences that AMCs will find meaningful. For instance, individuals with industry ties were significantly more likely than individuals without ties to think that COI policies unnecessarily hindered interactions with industry (p = .004), were ineffective at reducing harm to patients (p < .001), and were ineffective in reducing bias in medical education (p>.001).

**Conclusion:**

The survey is now free and publicly available for use by any institution. AMCs can use the results to update and refine policies, and to provide ongoing education regarding existing policies.

## Introduction

Relationships of physicians, researchers, and medical institutions with the pharmaceutical, device, and biotechnology industry contribute to the advancement of medical research and the development of life-saving technologies. However, these academic-industry partnerships are pervasive [1], and they can create conflicts of interest (COI) that may increase the risk of harm to patients, bias research and jeopardize public trust in academic medical centers (AMCs) [2–7]. In response, medical leaders [8], public officials [9], and advocates have recommended that AMCs adopt comprehensive conflict of interest policies to protect the integrity of patient care, medical education and research [10]. Researchers have also developed additional tools to address these concerns, such as a disclosure checklist to strengthen and standardize reporting of financial conflicts of interest. [11]

A majority of AMCs have now adopted written COI policies [12], which are periodically analyzed and rated [13, 14]. However, AMCs often have little information on the impact of their policies, including how they are perceived by their staff. In this paper, we discuss the development and results of piloting in three sites of a standardized survey instrument to fill this information gap. We studied (1) the feasibility of implementing this survey, (2) its ability to measure self-reported relationships with industry as well as knowledge of COI policies and respondent’s reactions to them and (3) its potential utility to COI administrators engaged in the ongoing challenge of revising and implementing new COI policies and practices.

## Materials and methods

### Survey goals and development

Our goal was to develop a freely available, user-friendly survey to measure perceptions and effectiveness of COI policies at AMCs. Additionally, information from the survey could assist institutional COI compliance leaders in effectively designing and implementing policies, as well as to evaluate them for improvement. To be clear, the goal of the quantitative analysis is not to make comparisons across the study sites or to draw generalizable conclusions about COI based upon survey responses. Rather, the goal of this analysis is twofold: 1) to provide evidence that the survey is sensitive to differences that AMCs will find meaningful; and 2) to provide preliminary descriptive pilot data that might be used in future investigations with more sites.

Community Catalyst, a non-profit health advocacy organization, established a “COI Policy Effectiveness Work Group” in June 2012 and regularly met with members to design the survey based on institutional compliance leader priorities. The Work Group included national experts on COI policy and compliance, and was chaired by LZ. The members were recruited from among leaders in the Association of American Medical Colleges’ Forum on Conflict of Interest in Academe. One member is a Dean and the others are Directors or former Directors of Offices of Compliance, Ethics, and/or Conflicts of Interest. To ensure universal applicability across medical schools and AMCs, the survey questions covered a range of universally applicable compliance-related concerns, while still allowing institutions to tailor for alignment with their own policies and culture. The survey needed to be brief and accessible via a widely available survey platform (Research Electronic Data Capture, REDCap [15]), and user-friendly to deploy and to analyze.

The content of the survey was developed by conducting a literature review and by seeking the input of the Work Group experts. A draft was refined by gaining interactive feedback from group members. The survey was further refined, both in format and in content, by testing it with selected volunteers through cognitive interviews with eight experts in the field to evaluate their cognitive processes as they completed the survey. Specifically, we assessed participants’ comprehension of instructions and survey questions, in addition to testing skip patterns and other survey organizational structures. We asked them about the deliberations they used to answer questions. Additionally, we asked about any missing survey components that they felt needed to be added [16]. We conducted interviews to the point of data saturation (n = 8) and then coded them for thematic content related to survey improvement. These themes were then carefully reviewed by MH, WW and SR, and all the themes were addressed in the final survey revisions. Finally, the survey was tested on approximately 50 people across the three pilot institutions to ensure online functionality.

The survey addresses domains which were identified as priorities by the Work Group experts who recommended that the instrument should be able to assess knowledge and awareness of COI policies; determine whether policies were being followed; assess perceptions of the value and/or burden of policies; and provide information to improve policies and implementation. Collecting some demographic information was also necessary for purposes of interpreting the results and planning follow-up interventions such as education. The domains and the specific elements of the survey are described in Table 1.

**Table 1:** Elements of “The COI Policies Survey”.

Section of Survey	Elements
Cover Sheet	• Statement of purpose by institution • Statement that responses are anonymous • IRB approval or waiver language
Demographic Information	• Professional role • Specialty • School or department • Years since finished terminal degree
Frequency and types of interactions with industry (self-report)	• Receipt of payment or compensation for food, advisory board service, travel, CME conferences, speaking, consulting, etc. (17 categories) from the drug, device or biotech industry • Number of contacts with drug or device sales representatives in last 30 days
Knowledge of Policies	• Awareness of institutional COI policies • Awareness of specific listed COI policy domains • Interest in more information on specific list policies
Perceptions of the effectiveness or burden of specific COI policies	• Assessment of actual COI policies at the institution (summarized) as: satisfactory, overly restrictive, needs to be strengthened • Perceived impact of these policies along six dimensions (e.g. reducing patient risks, hindering collaboration with industry, etc.)
Perception of colleague frequency of industry interactions	• Estimated percentage of faculty in respondent’s department who have interactions with industry

### Process for piloting

The survey was piloted April 2014 through January 2015 in three AMCs (n = 1578). The institutions varied in size, type (public/private, medical school/teaching hospital) and overall policy strengths, as rated by the American Medical Student Association [13] and the Institute on Medicine as a Profession [14]. All sites surveyed medical school faculty and administration. Site 3 also included residents, and Site 2 included additional faculty from dental and veterinary schools. Each site formed an implementation team and secured the support of institutional leadership. All institutions received an exemption from their Institutional Review Board (IRB), given that this was an anonymous survey with “minimal risk” intended to improve the quality of COI programs. No financial incentives were provided to respondents. In email messages from institutional leaders distributing the survey to staff and other recipients, participation in the survey was encouraged. Participants could opt-out of the survey by declining to follow the hyperlink to the survey, or by declining to submit their results after initiating the survey. Participants could also opt out of specific questions by declining to submit an answer. Consent to participate was implied by recipients’ conduct to complete and submit the survey. Between 70% to 90% of recipients at the three sites chose not to submit the survey, and thus opted out.

The electronic survey was supplied, allowing each site to easily upload the survey to their internal institutional REDCap accounts, and to customize their COI policy summaries and details about certain questions to align with their institution’s policies. Each site developed an implementation strategy, including who should receive the survey (e.g. faculty, fellows, residents, researchers, nurses, students, etc.) and how it should be disseminated (e.g. as a link in an email or on an intranet site). Each site collected and analyzed their results. SR, WW and MH were available to help sites through the process and to answer general questions. Given that the goal of this project is to test the feasibility of implementing the survey at the institutional level, minor changes to the survey were encouraged.

### Qualitative methodology: Survey implementation

WW, SR and/or MH conducted two semi-structured interviews on the implementation, preliminary results and possible implications with staff who fielded the survey at each site. These interviews were audio-recorded, with verbal permission provided by all parties. The interviews were transcribed and coded for content themes by WW and MH, and discrepancies were resolved in consensus meetings that also included SR.

### Quantitative methodology: Survey results from pilot sites

Descriptive statistics were calculated on the survey results using SAS v9.2 [17]. Overall response summaries were examined as well as differences by those who had any ties to industry at all, any “Group A” ties or any “Group B” ties. Group A financial ties include industry-provided free or reimbursed food; supplies; drug/medical samples; travel; entertainment; or subsidized admission to CME. Group B financial ties include industry financial support for speaking at CME or non-CME events; consulting; commercialization of intellectual property; participating in an endpoint adjudication committee; or participating in research. Based on the literature and their experience with AMC policies, the Work Group recognized these two sets of relationships are treated somewhat differently by AMCs. Group A relationships are transfers of value to physicians and are not compensation for services provided. Institutions generally place substantial restrictions on Group A financial relationships because they are more casual relationships whose value to providers is outweighed by their risks to patient safety, medical education and scientific integrity.

Group B relationships with industry are seen as more necessary for the conduct of research and development, which is reflected in more nuanced policies that allow for the relationships but aim to ensure that relationships do not adversely affect the integrity of research, medical education or patient care. The exception in this group is industry speaking engagements, especially for non-CME events, which present greater risks of introducing bias, as reflected in more restrictive policies at many AMCs. [13, 14] Group B relationships are also more significant in terms of time and direct pecuniary gain associated with services or intellectual contribution and tend to require a written contract.

Comparisons of these groups were performed using either a chi-square test or a T-test (or its non-parametric analogues where appropriate). Multivariate modeling was not planned or conducted given the goals of this study, described above.

## Results and discussion

### Survey distribution and response rates

A total of 1578 faculty and staff responded to the survey at the three sites. The response rate varied from 30% at Site 1 (n = 958), to 15–20% at Site 2 (n = 446) and 10–30% at Site 3 (n = 125). The response rates for Sites 2 and 3 are estimates, given that the denominator is not easily determined, based upon the method of survey deployment (e.g., a survey link was posted on an internal website). Site 1 used a method of direct emails to potential respondents, so the response rate was more precise. The distribution details and response rates are described in Table 2.

**Table 2:** COI survey distribution and responses.

**Distribution of Survey**	Site 1	Site 2	Site 3
Number of faculty receiving survey	3,220	3,092	1,200*
Distribution method	Emailed to faculty using individualized links	Emailed to faculty using single link	Link to survey on Intranet provided after COI disclosures; Emailed as a single link to Residents
Number of reminders	2	2	0
**Respondents**			
Number of respondents	958	446	125*
Percentage of distributed surveys completed	30%	15–20%**	10–13%*
No. specialties represented	35	29	18

* Includes both faculty and Residents. 107 out of 800 faculty/staff completed the survey, for response rate of 13.4%; 18 of 400 Residents completed the survey, for a response rate of 4.5%.

** An estimated 1,000 adjunct faculty were included as part of the total 3,092 faculty who received the survey. Since some but not all adjuncts are covered by the COI policies, the effective response rate for covered faculty may be as high as 20%, but this cannot be determined due to the anonymous nature of the survey.

It is possible that those who chose not to respond might disproportionately have ties with industry. However, many who have relationships with industry did respond and the survey allows them to comment on how they perceive the impact of the policies, both positive and negative. Comments submitted by respondents also indicated that they appreciated this opportunity for feedback, which was valued by the compliance leaders and was not used for sanctions.

Site 1 used the personalized email distribution function in REDCap, which still allows for anonymity. The message appeared as a personal email from the compliance director, but it could not be emailed or forwarded by other leaders. Reminders are directed only to those who have not responded. Site 2 distributed the survey via a link in personal emails from department leaders, and sent two non-targeted reminders. The compliance staff delegated the decision concerning who should receive the survey to department leaders, which created challenges in targeting. Site 3 distributed the survey by directing faculty/staff to a link at the end of the intranet page where they completed mandatory annual online COI disclosures. The lower response rate may indicate potential respondents did not want to complete the survey after spending time on the disclosure form. In addition, reminders could not be sent.

Based upon the interviews with the site survey administrators, the overall level of effort to implement the survey and analyze the results at each site was an estimated 35 hours. Site teams typically included the compliance director, an associate director, a coordinator and a data analyst. Overall, the survey deployment and administration was low-burden, as described in the content themes detailed below.

### Qualitative results: Survey implementation

#### Implementation

Gaining support from a wide range of institutional leaders and faculty was seen as critical to survey implementation and response rates. Teams found REDCap easy to use for customizing the survey content, launching the survey and analyzing results, and they used their own institutional on-site administrative REDCap support.

#### Utility of the survey process and results

Respondents represented a broad cross-section of departments, medical specialties, and years in practice, which site leaders thought was similar to the populations sampled.

All site leaders desired to benchmark their constituencies’ knowledge and views of institutional policies and thought the survey was well received. It gave respondents an important opportunity to provide feedback, seen as instrumental to identifying next steps for policy improvement. There was some concern about lower response rates in the sections on perceptions of the impact of specific policies, which could reflect the length of that section, or that respondents did not have adequate knowledge of the policies to respond at that level of detail. Overall, the sites recommended implementation of the survey tool at other institutions. One leader stated: “The value of this [survey] cannot be overestimated.”

A preliminary quantitative analysis by site found that a majority of respondents rated specific policies as “satisfactory,” which site leaders found gratifying. One stated: “By and large there was a consensus that favored the policies in their current form. But I was glad to receive some negative comments, because it gives more credibility to the survey.” The majority of respondents at each site had no financial ties with industry, which was seen by leaders as consistent with internal disclosures to the institutions.

Site leaders reported that the survey gave them a more valid picture of people’s perspectives, since they more commonly interact with professionals dissatisfied with COI policies and/or those with industry relationships. One leader thought the survey was also superior to the “typical” model of relying on leadership and focus groups for feedback. In addition, it addresses industry ties not included in standard internal disclosures and thus “surfaced some issues, such as meals at off-site meetings that may need to be addressed down the line.”

### Quantitative results: Survey results from pilot sites

#### Characteristics of respondents

Respondents across sites were predominantly physicians (64.5%), researchers (18.3%), professors (12.6%), and institutional leaders (chair, chief of service) (4.5%). Clinicians were from 33 different specialties, mostly highly represented in pediatrics (8.6%), internal medicine (8.6%), anesthesiology (8.1%), and radiology (6.7%). There was a good distribution of respondents by years since completing the terminal degree, with most in the 11–20 year bracket (27.4%) and the 21–30 year bracket (27.8%). See Table 3 for more details.

**Table 3:** Respondent demographics (n = 1578).

**Industry Relationships**	**Total Sample (n)**	**Total Sample (%)**
Has Any Industry Ties—Yes	656	41.6%
Has Group A Industry Ties–Yes*	582	36.9%
Has Group B Industry Ties–Yes**	344	21.8%
**Professional Role (not mutually exclusive)**	**Total Sample (n)**	**Total Sample (%)**
Physician	1018	64.5%
Researcher	288	18.3%
Professor	199	12.6%
Institutional Leader	71	4.5%
**Clinical Specialty (among Clinicians, n = 1017)**	**Total Sample (n)**	**Total Sample (%)**
Pediatrics	87	8.6%
Radiology	68	6.7%
Internal Medicine	80	7.9%
Hematology & Oncology	63	6.2%
Cardiology	51	5.0%
Anesthesiology	82	8.1%
**Years Since Completing terminal degree (e.g., MD, PhD, NP)**	**Total Sample (n)**	**Total Sample (%)**
0–5 years	127	8.4%
6–10 years	195	12.9%
11–20 years	413	27.4%
21–30 years	419	27.8%
31–40 years	289	19.2%
41+ years	65	4.3%

*Group A: industry-provided free or reimbursed food; supplies; drug/medical samples; travel; entertainment; or subsidized admission to CME events.

**Group B: industry financial support for speaking at CME or non-CME events; consulting; commercialization of intellectual property; participating in endpoint adjudication committee; or participating in research.

Note: An individual physician who indicated that they have any industry ties may have both Group A and Group B financial relationships.

#### Knowledge of COI policies and desire for further education

Over 90% of respondents were aware that COI policies exist at their institutions. There was variation, however, in their awareness of specific policies: 77.5% on acceptance of gifts, food or entertainment; 75.8% on managing conflicts of interest in research; 71.3% on endorsements/promotional statements; 58.9% on participation in industry sponsored events; 57% on industry funding for CME; 57% on ghostwriting; and only 21.8% aware of policies on restrictions on access provided to sales representatives, and 22.6% on recruiting patients for clinical research. Some open text comments also indicated a lack of knowledge and/or understanding of the policies. One site leader concluded that more education is needed and could be offered in mandatory online education, using “bite-sized components summarizing key principles”.

A majority of respondents (58.4%) reported no industry financial ties; 36.9% reported “Group A” ties (free or reimbursed food, supplies, samples, travel, entertainment, or support for CME); 21.8% reported “Group B” ties (industry support for CME events; consulting; commercialization of intellectual property; participating in endpoint adjudication committee; or participating in research). See Table 3.

Across both Group A and B, the most frequently reported financial ties were for meals (19.8% outside the workplace and 13.4% inside the workplace); consulting (11.8%); payment for services on advisory boards (9.8%); research (9.3%); speaking at non-accredited CME meetings (7.2%); speaking at accredited CME meetings (5.6%); and travel to professional meetings (5.3%). See Table 4 for details.

**Table 4:** Description of respondents’ financial ties with industry (n = 1578).

**Group A Ties (Received in past year)**	**Total Sample (n)**	**Total Sample (%)**
None	996	63.1%
Food/beverages outside workplace	312	19.8%
Food/beverages inside workplace	212	13.4%
Payment for service on a scientific advisory board/board of directors	154	9.8%
Reimbursement for travel to professional conferences	83	5.3%
Office supplies	71	4.5%
Reimbursement for other travel	59	3.7%
Free drug samples	41	2.6%
Free/subsidized admission to meetings or CME conferences (not as speaker)	13	0.8%
Free tickets to cultural or sporting events	4	0.3%
Other	45	2.9%
**Group B Ties (Received compensation in the past year)**	**Total Sample (n)**	**Total Sample (%)**
None	1234	78.2%
Consulting	186	11.8%
Participating in research supported by industry	146	9.3%
Speaking at non-accredited CME activities	113	7.2%
Speaking at accredited CME event	89	5.6%
Participating in end-point adjudication committee or data safety monitoring broad	39	2.5%
Commercialization of intellectual property	35	2.2%
Board of directors	5	0.3%
Other	15	1.0%

Note: The above categories of respondents with particular ties with industry are not mutually exclusive.

#### Faculty perceptions of COI policies

**Fig 1:**
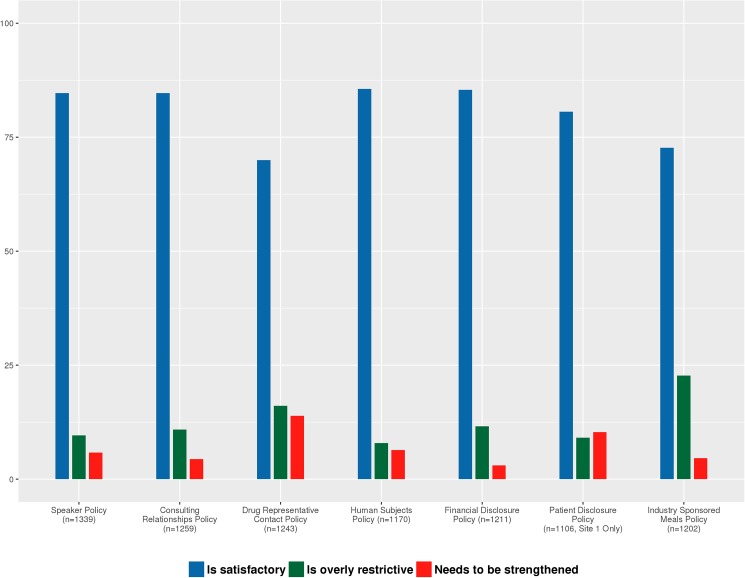
Respondents’ satisfaction with their institution’s COI policies.

The vast majority of respondents (85%) were satisfied with their institution’s policies on financial disclosure; human subjects’ protections; and industry support for speaking or consulting. A large majority (80%) were satisfied with the policy on disclosure to patients (Site 1 only). Satisfaction was lowest across all sites for policies on industry payment for meals (72.7%); and restricting access of industry sales representatives (70%). See Fig 1 for details.

Six follow-up questions on each policy measured perceptions of specific impacts. While response rates were lower for these follow-up questions, the results were nevertheless consistent with the results on overall satisfaction with policies. 60–67% of respondents perceived positive impacts of five policies (speaking; consulting; financial disclosure; sales representative access; and human subjects’ protections) in reducing harm to patients or research subjects, to bias in medical education and to research validity, while only 34–37% found these policies to unnecessarily hinder interactions with industry or hinder commercialization of intellectual property. Rates for perceived positive impacts were somewhat lower for the policies on meals and restricting access to sales representatives, while perceptions of negative effects were somewhat higher. See Table 5 for more details.

**Table 5:** Respondent perceptions of impact of selected COI policies (%).

	Speaker Policy		Consulting Relationship		Drug Rep Access		Financial Disclosure		Industry provided Meals	
	Mean	SD	Mean	SD	Mean	SD	Mean	SD	Mean	SD
**Reduces risk of harm to patients**	60.8	±26.7	60.8	±25.4	57.8	±28.2	62.2	±26.6	51.8	±29.6
**Reduces risk of harm to research subjects**	62.0	±25.4	61.9	±25.3	58	±25.6	63.6	±26.0	52.1	±30.2
**Protects validity of research data**	65.4	±25.1	63.3	±25.0	59	±26.2	64.8	±25.2	54.7	±30.1
**Reduces risk of bias in medical education bias**	63.8	±25.8	62.5	±25.1	60.9	±27.6	63.8	±26.0	53.3	±30.1
**Unnecessarily hinders interactions with industry**	36.1	±28.1	36.3	±26.9	37.9	±30.1	33	±27.9	40.4	±30.9
**Unnecessarily hinders IP commercialization**	35.3	±26.5	35.8	±26.6	34	±28.5	34.4	±28.0	32.9	±29.0

#### Differences in perceptions of “restrictiveness” of policies between those with and without financial ties to industry

We compared perceptions of the six to seven selected policies between those with any financial ties with industry and those without, as well as those in the “Group A” and “Group B” lists. Participants were asked if each policy “is satisfactory,” “is overly restrictive” and “needs to be strengthened.” While the majority of respondents considered policies to be satisfactory, the survey was able to detect significant differences in perceptions of restrictiveness (p-value < .001 in nearly all cases) between those with any ties and those without ties. (See Table 6 for more details.) This also held true when we distinguished between those with ties in the “Group A” and “Group B” lists. The survey was also sensitive to differences in the types of ties of respondents.

**Table 6:** Respondent perception of selected COI policies (%) by those with and without financial ties to industry.

	Speaker Policy (n = 1339)					Industry Provided Meals Policy (n = 1202)				
	No Financial Ties		With Financial Ties			No Financial Ties		With Financial Times		
	Mean	SD	Mean	SD	p-value	Mean	SD	Mean	SD	p-value
**Unnecessarily hinders interactions with industry**	32.4	±26.3	41	±29.6	0.004	35.4	±29.3	45.4	±31.6	<0.001
**Reduces risk of harm patients**	65.4	±24.9	54.8	±27.8	<0.001	57.8	±28.0	45.9	±30.0	<0.001
**Reduces risk of bias in medical education bias**	66.8	±24.2	60.1	±27.2	<0.001	59.5	±28.0	47.3	±30.8	<0.001

We also analyzed the follow-up questions for each policy by ties with industry. The differences between those with and without relationships were significant in most cases and the pattern was consistent—i.e. those with industry ties were significantly more likely than those without industry financial ties, to think that policies had negative effects (unnecessarily hindered interactions with industry and commercialization of intellectual property).

In the final survey question, respondents (n = 901–938) estimated the percentage of faculty that had six specific ties with industry. Respondents estimated that colleagues had on average twice as many ties as the results shown in the self-report sections of the survey.

## Conclusions

The results of this project demonstrate that surveying the target constituencies of COI policies at AMCs is both feasible and valuable. The COI administrators at each of these AMCs reported that the survey was low-burden. More importantly, the survey provided important data for these AMCs to evaluate their policies—something that is currently not systematically done among AMCs in the United States. Given that the survey is developed, free and ready to use in a format commonly supported by AMCs (REDCap) [14], the up-front costs to AMCs to utilize this tool have been significantly minimized.

AMCs can customize and implement this survey to gather information that is useful to help target and update ongoing education surrounding existing COI policies, to refine COI policies based on staff perceptions, and in some cases to gather evidence as to whether some of their policies are being adhered to. Data gathered with this survey will provide a significant improvement over what AMCs currently have at their disposal, which is usually feedback from people unhappy with specific policies and procedures. The survey provides a broader range of perspectives, including from those who have financial ties and from those who do not. Each institution that administers the survey would have to make its own individualized assessment of whether their staff’s awareness of, or support for, a COI policy affected the policy’s impact. Our expert Work Group recommended that the survey should measure awareness and support of policies, because hospital administrators may have skewed perspectives regarding employee perception of their policies, given that they hear most often from the few staff with extensive interactions with industry, some of whom may be running afoul of the restrictions on those interactions. However, beyond measuring awareness, impact on patient care, education, and research, and perception of policies, the survey also includes sections on self-reported relationships with industry. Thus, it could be administered periodically to measure changes in those relationships and compliance, perhaps as a pre and post-test of newly introduced policies or education on policies. Yet simultaneously measuring awareness and support for the institution’s policies remains critical, because changes in reported interactions with industry could be the result of external forces as well.

Our results are significantly different on some measures than that reported in a national survey by Campbell and colleagues in 2009 [1], which found that 83.8% of primary care and specialty physicians had relationships with industry. By far the highest percent of reported relationships was for industry supplied gifts (70.8%), food/beverage (70.6%) and drug samples (63.8%). However, that study did not stratify results by practice site, and AMCS in general and our pilot sites in particular, have very restrictive policies on acceptance of gifts, food/beverages and samples, many implemented since 2009. This is reflected in our results: only 13.4% (inside the workplace) and 19.8% (outside the workplace) received food or beverages from industry and only 2.6% received samples. Indeed, this contrast is a good indication that the pilot site policies may be having an impact in these areas, even though compliance could still be improved for food/beverages. Under policies at two of our pilot sites staff cannot accept samples or gifts of any kind from vendors, and the third site has significant restrictions on both as well. Where policies in our pilot sites are less restrictive and nuanced, such as speaking and consulting, the percentages of respondents with relationships are comparable. Campbell et al found that 6.7% were paid for consulting, compared to 11.8% in our sample (likely due to the role of research at AMCs), and Campbell et al found that 8.6% were in speaker’s bureaus, nearly identical to our results, where 9.3% were paid for speaking at non-accredited CME activities and 7.2% for CME accredited events.

While a low response rate was identified as a potential concern by Sites 2 and 3, those rates are typical for physician surveys, especially when no financial incentive is provided [18]. We recommend that people use a particular feature in REDCap that allows for personalized, direct emails, while allowing for anonymous responses. Furthermore, we encourage AMCs to gain the support of institutional leaders, who can best impart the importance of this survey to their employees and faculty members.

The in-depth analysis of perceptions of policies by those with and without ties shows the survey is able to distinguish significant differences between groups of faculty. These data can assist institutional leaders in further understanding faculty perceptions and tailoring effective responses. For instance, the results could surface the need for education in particular departments or on specific policies, as well as the need to better enforce some policies or revise others.

The survey is now freely available (see S1 Appendix) for use by any institution to help assess COI policy compliance, awareness and perception of policies. An internal evaluation tool like this may be especially valuable in light of the CMS’ Open Payments program, which publicly reports payments and transfers of value to physicians and teaching hospitals by the drug, device and biologic industry [19].

## Supporting information

S1 Appendix Survey Access Information. (DOCX)
