# Supplementary material for: A new survey to evaluate conflict of interest policies at academic medical centers
Source: PLoS One. 2017 Mar 15;12(3):e0172472. doi: 10.1371/journal.pone.0172472 (PMC5351836; doi:10.1371/journal.pone.0172472)
Supplement: S1 Appendix — (DOCX) [file pone.0172472.s001.docx]

**SI Appendix: Survey Access Information**

A generic version of the “Conflict of Interest Policies Survey” can be accessed at <https://redcap.ccf.org/redcap/surveys/?s=Cack2cK9I6>

The REDCap data dictionary for the generic survey, which can be easily adapted, can be obtained upon request from: Dr. Rose, roses2@ccf.org.
